# The effect of education and supervised exercise vs. education alone on the time to total hip replacement in patients with severe hip osteoarthritis. A randomized clinical trial protocol

**DOI:** 10.1186/1471-2474-14-21

**Published:** 2013-01-14

**Authors:** Carsten Jensen, Ewa M Roos, Per Kjærsgaard-Andersen, Søren Overgaard

**Affiliations:** 1 Orthopedic Research Unit, Dept. of Orthopedic Surgery and Traumatology, Odense University Hospital, 29, Sdr. Boulevard, DK-5000, Odense C, Denmark; 2 Institute of Clinical Research, University of Southern Denmark, Odense C, Denmark; 3 Musculoskeletal Function and Physiotherapy, Institute of Sports Science and Clinical Biomechanics, University of Southern Denmark, Odense C, Denmark; 4 Sector for Hip and Knee Replacement, Dept. of Orthopedics, Vejle Hospital, Vejle, Denmark

**Keywords:** Exercise therapy, Education, Osteoarthritis, Hip, Hip replacement

## Abstract

**Background:**

The age- and gender-specific incidence of total hip replacement surgery has increased over the last two decades in all age groups. Recent studies indicate that non-surgical interventions are effective in reducing pain and disability, even at later stages of the disease when joint replacement is considered. We hypothesize that the time to hip replacement can be postponed in patients with severe hip osteoarthritis following participation in a patient education and supervised exercise program when compared to patients receiving patient education alone.

**Methods/design:**

A prospective, blinded, parallel-group multi-center trial (2 sites), with balanced randomization [1:1]. Patients with hip osteoarthritis and an indication for hip replacement surgery, aged 40 years and above, will be consecutively recruited and randomized into two treatment groups. The active treatment group will receive 3 months of supervised exercise consisting of 12 sessions of individualized, goal-based neuromuscular training, and 12 sessions of intensive resistance training plus patient education (3 sessions). The control group will receive only patient education (3 sessions). The primary end-point for assessing the effectiveness of the intervention is 12 months after baseline. However, follow-ups will also be performed once a year for at least 5 years. The *primary* outcome measure is the time to hip replacement surgery measured on a Kaplain-Meier survival curve from time of inclusion. *Secondary* outcome measures are the five subscales of the Hip disability and Osteoarthritis Outcome Score, physical activity level (UCLA activity score), and patient’s global perceived effect. *Other* measures include pain after exercise, joint-specific adverse events, exercise adherence, general health status (EQ-5D-5L), mechanical muscle strength and performance in physical tests. A cost-effectiveness analysis will also be performed.

**Discussion:**

To our knowledge, this is the first randomized clinical trial comparing a patient education plus supervised exercise program to patient education alone in hip osteoarthritis patients with an indication for surgery on the time to total hip replacement.

**Trial registration:**

NCT01697241

## Background

Osteoarthritis (OA) is the most common joint disease and a major contributor to pain, decreased physical function and decline in health-related quality of life [1]. Its incidence and prevalence will increase in the coming years due to the ageing of the population [2]. Already, the incidence of total hip replacement in Denmark, has increased from 75 per 100,000 in 1995 to 170 per 100,000 in 2009 [3], a development likely to continue as joint replacements are generally recommended at later stages of the disease and therefore, commonly experienced as one gets older. Such a development has socio-economic consequences, and thus, calls for potential non-surgical treatment alternatives, especially those which are effective in improving physical function and reducing pain to a level where health-related quality of life is restored, and the need for total joint replacement is at best eliminated, or at least, postponed.

Within the last decade, it has become well established that exercise reduces pain and improves function in patients with knee OA [4-12]. A recent meta-analysis on supervised exercise interventions showed that exercise had moderate effect on pain and self-reported physical function in patients with mild to moderate knee OA [11], and another meta-analysis found similar effects in those waiting for knee replacement [4]. Supported by these meta-analyses, exercise is recommended in clinical guidelines for the management of knee OA across the severity range of the disease [10]. Conversely, evidence to support exercise as part of the management of hip OA is lacking, primarily due to the absence of hip-specific studies [6,9,13]. Randomized Controlled Trials (RCTs) of exercise specific to hip OA are one of the top research priorities of the European League against Rheumatism (EULAR) [14].

Exercise treatment could potentially be used to postpone hip replacement surgery. Although, the optimal type of exercise is yet to be determined. An individualized approach to exercise prescription is needed based on assessment of impairments [10]. In addition, exercise classes are more effective in reducing pain than home-based programs [15], and both the progression and intensity should be carefully controlled as they are the strongest factors affecting functional outcome [16,17]. Hence, an exercise intervention aimed at postponing or eliminating surgery should be individualized, class-based, progression-controlled, and feasible for patients with severe hip OA.

A program based on neuromuscular principles was found to be feasible in elderly patients scheduled for hip and knee replacement, and it complied with the above recommendations for exercise prescription [18], while intensive resistance training has proven effective in hip patients in the early postoperative phase [19]. To mimic performance enhancement in sports, and possibly to boost the effect of the intervention, both exercise modalities will be used in this trial.

In summary, more evidence to support the use of exercise as non-surgical treatment in the management of patients with severe hip OA is needed, and its effect on the time to hip replacement surgery is largely unknown.

### Specific objective and hypothesis

The *primary* aim is to test the hypothesis that patients with severe hip OA are more likely to postpone hip replacement surgery following participation in a patient education and supervised exercise program when compared to patients receiving patient education alone. The *secondary* aim is to examine if self-reported musculoskeletal health defined as pain, physical function, physical activity and quality of life (HOOS), physical activity level (UCLA activity score), and the patient’s global perceived effect (GPE) improve more in the education and exercise group. *Other* measures include pain after exercise, joint-specific adverse events, exercise adherence, general health status (EQ-5D-5L), mechanical muscle strength and physical performance tests. A cost-effectiveness analysis will also be performed.

## Methods and materials

### Study design

A prospective, blinded, parallel-group, multi–center trial (2 sites), with balanced randomization [1:1], in accordance with CONSORT (Consolidated Standards of Reporting Trials) guidelines [20].

### Participants and recruitment procedures

Patients aged 40 years and older with an indication for total hip replacement (THR) for hip OA will be consecutively recruited from the outpatient clinic at the Department of Orthopedic Surgery and Traumatology, Odense University Hospital, and Vejle Hospital, Denmark. The inclusion and exclusion criteria are listed in Table 1. The indication for THR will be based upon symptoms, a clinical examination and radiographic findings [21]. Although, defining the cut points for symptoms and functional disability which indicate the need for a hip replacement is complex [22]. Data on multiple other factors such as home situation, co-morbidities, and marital status will be collected at baseline. An independent radiologist will assess the stage of hip OA severity using the Kellgren-Lawrence grading scale [23].

**Table 1:** Criteria for participants in the study

**Inclusion criteria**	**Exclusion criteria**
40 years and older	Inflammatory joint disease
Indication for total hip replacement	Earlier ipsilateral proximal femur fracture
Residency within local municipality or willing to commute	Hip pain < 3 months
	Neuropathy or neuromuscular disease
	Malignant disease
	Diseases where a moderate level of physical exercise is contraindicated
	Inability to speak or read Danish
	Inability to participate for other reasons
	Refusal to participate

On initial contact, patients will be examined and an indication for hip surgery will be assessed by an orthopedic surgeon. Where all inclusion criteria and none of the exclusion criteria are met, the patients will be invited to participate and will receive verbal and written information about the trial (background, procedure, randomization, and potential risks). In addition, a standardized information video without technical or value-laden terms will be provided to patients to ensure impartiality between treatments and consistency at both hospitals. Information will be given in an undisturbed location within the outpatient clinic, and time will be made available for questions. Subsequently, the patients will receive the written consent form. Patients wishing to have some reflection time will be given a stamped addressed envelope, and contacted by the research nurse no later than 2 days after consultation. Patients who choose not to have reflection time will sign the written consent form, be randomized, and scheduled for the completion of baseline measurements.

**Figure 1:**
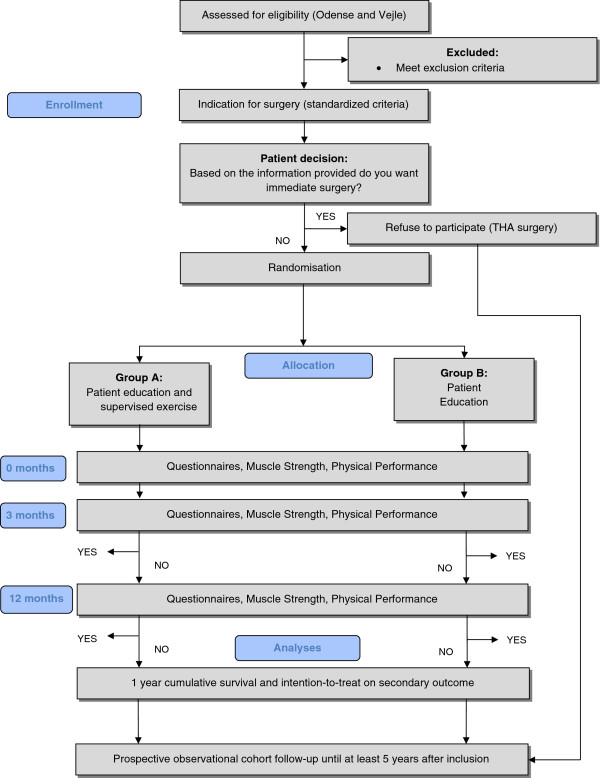
Study flow chart through-out the randomized clinical trial.

A flow diagram of the progress (enrolment, randomization, intervention allocation, and follow-up and data analysis) through the trial can be found in Figure 1.

### Randomization and allocation concealment

Block randomization will be computer-generated in two steps by a person (JL) not involved in the trial. At first, stratification by hospital will be performed. Next, patients will be allocated to one of the two interventions by a sequence of letters: A – referring to patient education plus supervised exercise, and B – referring to patient education alone. Block sizes will be balanced within 5 blocks of 20 patients in each strata. The allocation sequence will be concealed in sequentially numbered opaque, sealed envelopes. To prevent subversion of the allocation sequence, the name and date of birth of the participant will be written on the envelope. Envelopes will be opened only after the enrolled patients have completed all baseline assessments and subsequent appointments have been made based on treatment allocation.

### Blinding

Blinding to treatment allocation of patients, physiotherapists and nurses (health care providers) will not be possible due to the nature of the interventions. However, independent data collectors will be responsible for baseline and follow-up assessments, while all self-reported outcome questionnaires will be made available to the patients electronically, and responses entered into a database identified by identification numbers only. The data analyst (principal investigator, CJ) will be unaware of the treatment allocation as data will be analyzed using recoded identification numbers. The recoding will be performed by an independent person (JL).

### Interventions

The active treatment group (EDU+EX) will consist of patient education and supervised exercise. The control group (EDU) will consist of patient education only. Patients in group EDU+EX will receive two types of exercise, delivered on separate days. One type of exercise is class-based, individualized, goal-oriented NEuroMuscular EXercise training for patients who are scheduled for total joint replacement (NEMEX-TJR). The other type of exercise will be class-based, individualized, intensive Resistance Training (RT). Both groups will receive education, which will be administered on separate days to ensure that EDU+EX patients receive education independently from patients randomized to EDU.

#### The NEMEX-TJR exercise program

The aim of NEMEX-TJR training is to improve sensorimotor control and achieve compensatory functional stability. Functional, weight-bearing exercises are used in various positions, resembling conditions of daily living and more strenuous activities [18]. The program emphasizes neutral alignment of the hip, knee and foot, and although focus is on the affected leg, exercises are carried out with both legs. Progression is guided by the patient’s neuromuscular function [18] to accommodate the individual’s level of function and pain. The NEMEX-TJR program has previously been described in detail and is available for download as an additional file [18].

#### The RT exercise program

The aim of intensive resistance training is to improve functional capacity through improvements in muscle strength and power. Progression in these exercises is guided by load [24]. Thus, the quantity of muscle output and the stimulation of neural adaptation (neural drive) is emphasized. Multiple and single joint, non-weight-bearing exercises targeting isolated muscle groups are used. Each exercise is performed as three sets of 8–12 repetitions equal to 70-80% of 1 Repetition Maximum (RM) with time for rest equivalent to that of one set [25-27].

Each of the two types of exercise will be offered weekly during the 12-week intervention period in sessions lasting 60–70 minutes. Thus, the entire exercise intervention will consist of 24 sessions, and the patients will be admitted continuously into the class-based groups so each group consists of both novice and experienced participants. Exercise professionals who are experienced in the execution of both types of exercise will be responsible for the patient education and supervised exercise program.

Both types of exercise may provoke pain and patients will therefore be asked to rate their pain intensity (0=no pain, 10=worst possible pain) before and after each session on a visual analogue scale (VAS). In accordance with the NEXEM-TJR program the patients will be told that pain up to a level of 5 is considered “acceptable” after each training session. The day after training, their pain should subside to ‘pain as usual’ and not increase over time. ‘Pain as usual’ is defined as the pain level prior to exercise. If this does not occur, the level of training progression will be reduced [18].

#### The patient education program

The patient education program is designed to encourage the patient to actively engage in and take responsibility for the management and treatment of their hip OA. The program is called GLA:D (Good Life with Arthritis in Denmark), and is based on principles used in The Osteoarthritis Management Course developed in Sweden (BOA, Gothenburg, Sweden) [28]. It consists of three sessions, each with a duration of 90 minutes. The content of the program has been translated into Danish and found to be feasible. All physiotherapists will attend a course in delivering the GLA:D concept to ensure consistency. Sessions will be held in groups of up to 15 participants. The physiotherapists responsible for conducting the program will facilitate interaction and discussions between the patients. The education group will be similarly monitored to the supervised exercise group.

### Intervention and follow-up period

Assessments will be made at baseline, 3 and 12 months after randomization, and both treatment groups will have completed the intervention after 3 months [29]. After the intervention, patients will be encouraged to continue the exercise program unsupervised at home or in public fitness centers.

### Outcome measures

One primary outcome measure has been chosen to avoid problems of interpretation associated with multiplicity of analysis [30,31]. It will be supported by a range of self-reported secondary outcome measures.

### Primary outcome measure

Cumulative Survival analysis (Kaplan-Meier survival curve).

Cumulative survival is measured as the time in days without total hip replacement surgery since inclusion.

### Secondary outcome measures

#### The Hip disability and Osteoarthritis Outcome Score (HOOS 2.0)

HOOS is a patient-reported outcome measure with five subscales for pain, other symptoms, function in daily living, function in sport and recreation and health-related quality of life (QoL) [32]. A 5-point Likert-scale will be used and converted into a 100-point scale with zero indicating the worst possible health [33].

#### Physical activity level

The University of California Los Angeles activity score (UCLA activity score) is a 10-point Likert scale recommended and used extensively in similar populations [34,35].

#### Global perceived effect (GPE) score

In addition to pain and physical function, the assessment by the patient of a global perceived effect of the treatment is a recommended responder criterion [36]. Patients will be asked to rate possible change in their condition (pain, symptoms, ADL, sport and recreation, QoL, level of physical activity, treatment evaluation) since the initial administration (baseline) on a 7-point Likert scale. A 2-point change from ‘no change’ will be used as cut-off to categorize the response into: 1) better, 2) no change, and 3) worse, with ‘no change’ being the neutral response.

### Other measures

#### Self-reported exercise pain

Pain will be self-reported immediately before and after each exercise session using a VAS scale. Pain scores will be grouped into 0–2 indicating safe, > 2-5 acceptable, and > 5 high risk pain [18]. Self-reported pain after exercise will be used to rate the overall percentage of training sessions completed with acceptable pain, and to evaluate joint-specific adverse events (detailed later).

#### Exercise adherence

‘The extent to which a person’s behavior corresponds with agreed recommendations from a health care provider’ [37]. Adherence will be measured as the number of training sessions completed out of the expected 24 sessions, and will be analyzed separately for RT and NEMEX exercises. Excellent adherence will be defined as participation in 75% or more of the exercise sessions, good as 50-74%, moderate as 25-49%, and poor when participation in less than 25% of the sessions. Patients ceasing training or unwilling to continue will be referred back to the orthopedic department for an optional total joint replacement without having to enter into possible waiting lists.

#### Mechanical muscle strength

Isometric muscle strength (iMVC) for knee extensors, hip extensors, hip flexors and hip abductors using stabilized dynamometry.

#### Physical performance battery

Physical performance-based measures will include: 30-second repeated chair raise test (number completed), 20-meter fast-paced walk (time in seconds), 30-second single-leg knee bending (number completed), and Timed Up and Go (time in seconds).

#### General health status (EQ-5D-5L)

A utility index for use in health economics. It is based on a descriptive system that defines health in terms of five dimensions: Mobility, Self-Care, Usual Activities, Pain/Discomfort, and Anxiety/Depression [38].

#### Cost-effectiveness

This will be estimated as the ratio between the cost of the intervention and the effect it produces. The total cost will be estimated from register-based costing of primary care, secondary care (length of hospital stay, re-admission, visits at the emergency room and out-patient clinic) and patient’s out-of-pocket costs (transportation expenses and time spent on transportation and receiving health care). Quality of Life Adjusted Years (QALYs) will be used for the analysis and calculated using changes in EQ-5D-5L from baseline to the 12-month follow-up. Patient-reported values are weighted using Danish tariffs [39].

#### Poor compliance

Poor compliance is not uncommon in studies of non-surgical treatments [40]. Achieved exercise dose and reasons for poor-compliance will be recorded.

#### Joint-specific adverse events

Joint-specific adverse events will be determined as: 1) not attending a training session and/or ceasing training because of increased pain/problems in the index joint related to the training; and 2) self-reported pain level > 5 on the VAS scale after training. The reasons for not attending a session due to pain/problems related to training or to other factors will be recorded.

### Sample size

One year has been selected as a clinically relevant period to postpone surgery. The survival probability in the education plus exercise group at one year is estimated to be 0.80 and 0.60 for the education only group. Survival group differences will be tested using log-rank statistics. To achieve a statistical power of 80% (β=0.20), the estimated sample size for a two-sample comparison (days without surgery) of survivor functions (log-rank), a sample size of n=86 is needed in each group to detect statistically significant differences (for α=0.05). Allowing for no more than 15% drop-out, 200 patients in total will be recruited.

### Statistical analysis

All outcome measures will be checked for Gaussian distribution, and non-parametric data will be analyzed using a Mann–Whitney rank sum test [41]. The treatment effect will be analyzed using a random effects mixed linear model (repeated measures) [42]. Hence, correlated measurements from the same patient can be modeled. Model assumptions are checked by residual plots and the model includes the interaction between treatment and elapsed time, adjusted for baseline values. All analysis will follow the intention-to-treat principle [43]. However, subsequent analysis using last observation carried forward may be required. Finally, the number needed to treat (NNT) for a positive effect of treatment (no surgery after 1 year) will be analyzed. All statistical analyses are blinded and will be performed using Stata 11 software (StataCorp, TX, USA).

### Facilities, organization and timeline

Exercise professionals from the local municipal health agency will participate. The project utilizes already established exercise facilities within the municipality. This collaboration between several health care professionals enables us to conduct a clinically founded randomized trial based on evidence from several research areas. The use of a multi-center design facilitates recruitment, and possible implementation of a new treatment strategy. Inclusion of patients will commence 2013 and end 2014 and with the 12 month follow-up, the last patient will be followed-up in 2015. In the continuation of this trial, we intend to follow patients (prospective observational cohort study) to provide long-term results (Figure 1).

### Ethics

The trial complies with the Declaration of Helsinki and ethics approval has been granted by the Regional Ethics Committee of Southern Denmark, approval number S-20120109. The trial is registered with ClinicalTrials [44] (NCT01697241).

## Discussion

To our knowledge this is the first randomized clinical trial comparing the effect of a patient education plus supervised exercise program to education alone on the time to total hip replacement in patients with an indication for hip replacement surgery.

Such a trial is warranted because exercise as non-surgical treatment may help to postpone or eliminate the need for TJR without deterioration and also improve health-related quality of life through enhanced function and decreased pain. Hence, studying patients with this severe disease condition is clinically important. Further, the primary outcome measure ‘time in days without surgery’ was chosen because TJR should only be recommended once improvements cannot be achieved by other treatment strategies. Moreover, postponing or avoiding TJR is the most relevant outcome when it comes to: reducing health care costs providing the program is cost-effective, minimizing complications, and improving healthcare pathways. Finally, such a trial is also warranted because the recommendations for lower extremity exercises are based on studies of knee OA patients. Although exercise trials on patients with hip OA have been conducted, most of these studies have also included patients with coexisting knee OA, and results were not joint-specifically reported, or too few patients with hip OA were included, preventing valid conclusions to be drawn [6,9,12,15]. Consequently, evidence supporting exercise for patients with hip OA is needed [14].

The secondary outcome measures used in this trial are those recommended for use in clinical studies of OA, and include patient-reported outcome measures (PROs) for pain, physical function, physical activity, health-related quality of life, and global response to treatment [36]. The patients will be asked to rate their current hip condition at baseline, and at each follow up (HOOS questionnaire), but also to rate possible change in their condition since baseline (GPE questionnaire). The 3-month follow-up was chosen to identify possible improvements at the end of the intervention period. Three months is also a reasonably long timeframe for clinical improvements to occur in patients with long-standing hip pain, yet short enough to assume that patients would be able to recall their baseline condition. The 12-month follow-up was chosen to match the clinically relevant period for postponing hip replacement surgery.

As for the other measures, both muscle mechanical and physical performance-based outcome measures will be part of the evaluation for primarily three reasons. First, muscle mechanic outcomes provide information about the effectiveness of the exercise intervention in terms of improving muscle strength, and both muscle weakness [45] and muscle asymmetry [46,47] have been found to be present in hip OA patients. Second, physical performance-based outcomes provide information about the effectiveness of the exercise intervention in terms of improved function in tasks resembling daily activities. Third, muscle atrophy has been associated with functional limitations in aging individuals [48], and this trial is adequately powered to further explore such associations. Furthermore, measures of exercise-related pain, adherence, and joint-specific adverse events are essential for evaluating the feasibility of the exercise intervention program. Finally, the cost-effectiveness of non-surgical interventions for hip OA is needed [49]. Total costs during the first year will be compared with gains on the HOOS and the patient’s perceived effect (GPE), and the societal costs of the intervention per patient during the first year and the incremental qualify of life in years/days, as obtained from the EuroQol (QALY during the first year). The gain in health and the costs associated with the heath gain could possibly be used as a decision-making aid for optimal resource allocation.

A number of considerations went into designing the supervised exercise intervention in this trial. One consideration was how to prescribe an exercise program, where the level of progression gradually increases, without provoking persistent pain. The use of self-reported pain (VAS) immediately after each exercise session has previously proven to be a feasible way of adjusting the level of progression [18]. Hence, patients who experience recurrent high risk pain after exercise or whose pain either doesn’t return to ‘pain as usual’ or increases during the day following exercise will be asked to reduce the level of exercise to the previous level of progression.

Another consideration was selecting the type of exercises to be used in this trial. An exercise program based on neuromuscular principles has been shown to be patient- and goal-oriented and feasible for elderly patients scheduled for hip and knee replacement [18]. All exercises are individualized with progression guided by the patient’s neuromuscular function, and for each exercise, three levels of progression are available, meaning the program can be tailored to the individual’s pain level and function. It utilizes functional exercises and emphasizes neutral alignment of the hip, knee and foot to resemble conditions in daily life and more strenuous activities, without compromising the quality of the performance. Another exercise program using progressive high load (intensive) resistance training principles has been shown to be effective in elderly patients in the early postoperative phase [19] and elderly hospitalized patients [50]. Also, evidence of the efficacy of general pre-operative strengthening exercises exists [4], and strengthening exercises alone show moderate effect on pain and function in patients with OA [51]. Rather than using general strengthening exercises, the use of high load (70-80% of 1RM) in the current trial is based on evidence from systematic reviews, where the intensity of the training was the strongest factor affecting strength and functional outcome [16,17]. To be effective, exercises must target isolated muscle groups, be progressive [26,27], and possibly be combined with a more complete exercise program [51]. Both exercise programs in this trial are supervised, based on individualized goal-based patient treatment that allows the program to target the presenting musculoskeletal impairments that contribute to the patient’s symptoms and functional limitations. This approach aligns with the guidelines from EULAR, which recommend tailored treatment [14].

A final consideration was how to most effectively distribute the two types of exercises throughout the intervention period, and across each week. We decided to mimic performance enhancement in sports. As a result, neuromuscular and intensive resistance exercise sessions will be administered on separate days within the same week, so that separate session goals can then be pursued.

Optimal outcomes in population health require both efficacious treatments and adherence to those treatments. Adherence to health interventions is a complex issue, especially for individuals with chronic conditions [52]. The interaction between patients and providers in terms of verbal communication, physical interaction and empathy could affect the patient’s enthusiasm, adherence, and participation. Nevertheless, it can be enhanced by the use of supervised, class-format exercise sessions in the initial exercise period [10] combined with later ‘refresher or booster’ sessions [52,53]. Also, to minimize the provider effect, three physiotherapists will randomly interact and execute the training program at each center in this trial.

The internal validity of the trial may be affected by a negative performance bias in the group who receive patient education only, as they may feel neglected and missing out on active treatment. The exclusion criteria always limit the generalizability to other populations. For example, the results from this trial may not be generalized to an individual patient with hip OA who is not fit enough to take part in moderate-to-high intensity exercise. Conversely, the external validity is improved by the multi-center design, and the delivery of the interventions by multiple practicing community physiotherapists. Also, the intervention will be implemented for both genders, a wide age range, and in patients with severe hip OA. Hence, it is reasonable to assume that the program could benefit the general hip OA population. Lack of adherence is a potential confounding factor for the outcomes in this trial. Consequently, the number of sessions completed will be recorded, and patients will be asked specifically at follow-up points if they have initiated other treatments for their hip condition since the last examination [52].

## Conclusion

We have designed this randomized clinical trial with the main purpose being to compare the effect of education plus supervised exercise to education alone on the time to hip replacement surgery in patients with an indication for hip replacement surgery. The results will provide evidence for the effectiveness of supervised exercise in relation to the postponement of hip replacement surgery, and the cost-effectiveness of the intervention. It may also redefine current treatment strategies for patients with severe osteoarthritis of the hip.

The results will be submitted to a peer-reviewed international journal for publication irrespective of the outcome in accordance with the CONSORT guidelines for reporting of clinical trials [20,30].

## Abbreviations

ADL: Activities of Daily Living; EQ-5D-5L: EuroQol; GPE: Global Perceived Effect; HOOS: The Hip disability and Osteoarthritis Outcome Score; iMVC: Isometric Maximal Voluntary Contraction; NEMEX-TJR: Neuromuscular Exercise for patients eligible for total joint replacement; QoL: Quality of Life; RT: Resistance Training; TJR: Total Joint Replacement; UCLA: University of California Los Angeles activity score; VAS: Visual Analog Scale.

## Competing interests

The authors declare that they have no competing interests.

## Authors’ contributions

CJ and SO conceived the project and procured the project funding. CJ is leading the coordination of the trial and is responsible for drafting this manuscript. ER provided intellectual input on the design of the trial and provided the training of the study physiotherapists in administering patient education and neuromuscular exercise. All authors participated in the trial design, provided feedback on drafts of this protocol and read and approved the final manuscript.

## Pre-publication history

The pre-publication history for this paper can be accessed here:

http://www.biomedcentral.com/1471-2474/14/21/prepub
